# Urban sparrows respond to a sexually selected trait with increased aggression in noise

**DOI:** 10.1038/s41598-018-25834-6

**Published:** 2018-05-14

**Authors:** Jennifer N. Phillips, Elizabeth P. Derryberry

**Affiliations:** 1 Department of Ecology and Evolutionary Biology, Tulane University, New Orleans, LA 70118 USA; 2 Department of Biological Sciences, California Polytechnic State University, San Luis Obispo, CA 93407 USA; 3 Department of Ecology and Evolutionary Biology, University of Tennessee, Knoxville, TN 37996 USA

## Abstract

Animals modify acoustic communication signals in response to noise pollution, but consequences of these modifications are unknown. Vocalizations that transmit best in noise may not be those that best signal male quality, leading to potential conflict between selection pressures. For example, slow paced, narrow bandwidth songs transmit better in noise but are less effective in mate choice and competition than fast paced, wide bandwidth songs. We test the hypothesis that noise affects response to song pace and bandwidth in the context of competition using white-crowned sparrows (*Zonotrichia leucophrys*). We measure male response to song variation along a gradient of ambient noise levels in San Francisco, CA. We find that males discriminate between wide and narrow bandwidth songs but not between slow and fast paced songs. These findings are biologically relevant because songs in noisy areas tend to have narrow bandwidths. Therefore, this song phenotype potentially increases transmission distance in noise, but elicits weaker responses from competitors. Further, we find that males respond more strongly to stimuli in noisier conditions, supporting the ‘urban anger’ hypothesis. We suggest that noise affects male responsiveness to song, possibly leading to more territorial conflict in urban areas.

## Introduction

The acoustic adaptation hypothesis states that organisms will adapt their vocalizations to transmit best in their respective environments^1,2^. There are many studies supporting this hypothesis in natural soundscapes, with organisms changing the timing, amplitude, or frequency of vocalizations to maximize sound transmission to receivers^3^. More recently, research has shown that animals also adjust their signals to evolutionarily unprecedented anthropogenic soundscapes^4^, which can alter the acoustic window of optimal signal transmission^5^. This phenomena occurs across a variety of taxa, including mammals^6^, amphibians^7^, fish^8,9^, invertebrates^10^, and birds^11^. Bird song has the most accumulated evidence for an effect of anthropogenic noise on communication, with many species singing higher pitched songs in urban areas^11^.

Generated by machines – such as boats, cars, and industrial equipment – anthropogenic noise is typically loudest at low frequencies (<2 kHz), and often overlaps (i.e. masks) the lower frequency range of animal signals^12^. Masking can decrease the ability of receivers to detect or discriminate the information content of a signal^13^ and thus alter the behavioral response of the receiver. For example, masking noise decreases the ability of budgerigars (*Melopsittacus undulates*) and zebra finches (*Taeniopygia guttata*) to discriminate between different calls^13^. Furthermore, great tits (*Parus major*) are less efficient at discriminating low frequency songs in urban noise^14^. Another study shows that female grey treefrogs (*Hyla chrysoscelis*) took longer to orient and approach male signals in masking noise, and their detection thresholds increased^15^. Therefore, masking noise can even lead to ‘evolutionarily inappropriate responses’ by receivers^16^, resulting in fitness costs to the signaler, the receiver, or both.

As songs in birds are often used for mate attraction and territory defense, environmentally induced modifications to signals may pose fitness costs and benefits^17^. In the context of mate choice, fertile females typically prefer low pitch songs when they can hear them, suggesting sexual selection on lower song frequencies via mate choice; however, in noisy conditions, females respond more to high pitch songs^14,18^. In the context of male-male competition, territorial males of several species respond less to urban associated increases in song minimum frequency^11^. These findings suggest that males that produce songs with higher minimum frequencies are less competitive, at least in areas with lower noise levels^19^. Therefore, noise can affect how signals are perceived, and acoustic adaptation may sometimes work in opposition to the preference or competitive function of the signal.

Birds adjust not only pitch but also many other features of song to urban noise levels, but the functional consequences of these changes remains poorly resolved. A loss of bandwidth due to changes in minimum but not maximum frequency in noisy areas has been reported in many species^11,20^. Northern cardinals (*Cardinalis cardinalis*) and gray catbirds (*Dumetella carolinensis*) show a reduced bandwidth from changes in both minimum and maximum frequencies with increasing levels of noise^19^. City birds also change the rate or number of notes in noise, with some birds singing fewer notes^11,21^, or more of certain notes, like the twitter phrase of common blackbirds (*Turdus merula*)^22^. Acoustic adaptation theory posits that vocal signals are adapted to best travel in their given environment^1,2^. Thus, adjustments to bandwidth and trill rate in cities may be adaptations to transmit better in urban soundscapes, as slow trills and narrower bandwidths are less likely to degrade in ‘urban canyons’ (i.e., where buildings and other impervious structures become reverberating echo chambers)^23–25^, and narrow bandwidths require lower signal-to-noise ratios to be detectable^13^. However, variation in trill rate and bandwidth is also under sexual selection in the contexts of female mate choice and male-male competition^26–31^. Therefore, adjustments to trill rate and bandwidth via acoustic adaptation may interfere with a signaler’s ability to convey information to a receiver.

Broadband vocalizations of repeated notes (trills) are difficult to produce because many songbirds coordinate rapid vocal tract and beak movements to track dominant frequencies and filter out harmonics^32,33^. In birds with broadband trilled notes, there is a tradeoff between the rate of note production (trill rate) and note bandwidth. To sing slow trills, males can produce wide or narrow frequency bandwidths, because the timing between notes allows for mechanical movements of the bill, laryngeal muscles, and syrinx^34–36^. As a male increases his trill rate, timing between notes is decreased, and bandwidth is limited by mechanical constraints. A review across families of songbirds (Emberizidae, Cardinalidae, Fringillidae, and Passerellidae) found that this performance tradeoff forms a triangular distribution^37^, which has since been described in many other taxonomic groups^26,31,38^. How well a male can perform this tradeoff is called vocal deviation, and can be measured as the orthogonal deviation from an upper bound regression on this triangular distribution; a larger deviation score indicates lower performance, and a small deviation indicates higher performance^39^.

We lack an understanding of the communication function of vocal performance as measured by vocal deviation (hereafter, vocal performance) for sexual selection in the context of urban soundscapes. It is unknown whether this measure of vocal performance is a salient signal in areas of high anthropogenic noise, such as cities. Female birds and mammals prefer higher performance songs^26,40–42^. Males of various songbird species are able to discriminate between high and low performance^27,31^. However, no studies have considered selective pressures on vocal performance for signal transmission in conjunction with selective pressure for signals that inform receivers of signaler quality.

The Nuttall’s white-crowned sparrow (*Zonotrichia leucophrys nuttalli*; NWCS) is a good system in which to address this question because of previous work both on the function of vocal performance in male-male competition^27,43^ and on correlations between anthropogenic noise levels and variation in trill rate, bandwidth and vocal performance of their songs^28,44,45^. In previous studies, we found that male NWCS in both urban and rural areas respond more strongly to high performance than to much lower performance songs^27^. Further, males in rural areas respond equally to songs of similar vocal deviation, whether that vocal deviation is close to population average performance through increasing bandwidth or through increasing trill rate^43^. We do not know if this pattern of response holds in anthropogenic noise. In urban areas, NWCS adjust both trill rate and bandwidth in response to anthropogenic noise levels: males defending territories with higher than average noise levels (hereafter, noisier territories) produce songs with faster trill rates but narrower bandwidths than males on quieter territories and have lower performance songs^28,45^. Urban males also respond less to songs with narrower bandwidths^27,28^ yielding the hypothesis that noise-dependent adjustments of bandwidth and trill rate might have functional consequences, and that these consequences could vary with ambient noise levels.

Here, we test this hypothesis by measuring male response to variation in vocal performance as measured by vocal deviation across an urban gradient of noise, using NWCS breeding in San Francisco, CA U.S.A. We measure response to three stimulus treatments: 1) a high performance song with fast trill, wide bandwidth, 2) a lower performance song with fast trill, narrow bandwidth (typically found in noisier territories), and 3) a lower performance song with slow trill, wide bandwidth (typically found in quieter territories). We make several predictions about the importance and directional responses of males to vocal performance across noise conditions. First, we predict that males in noisier areas will not respond differently to wide bandwidth (high performance) and narrow bandwidth (low performance) songs, because noise masks low frequencies in the song, potentially making it difficult for males to detect differences in bandwidth. We predict that even in noisy conditions males will respond more to fast trill (high performance) than to slow trill (low performance) songs, as our urban birds are not found in ‘urban canyons’ which can mask fast trills. Based on studies finding stronger response to playback in cities^46–50^, we also predict that males will respond more strongly to song playback in noisier areas. Finally, we predict that as noise increases, discrimination between song types will be reduced, consistent with studies on other avian species^51^.

## Methods

### Song recordings and stimuli

We recorded songs in San Francisco using a Marantz PMD 661 digital recorder, Sennheiser omnidirectional microphone, and Saul Mineroff SME-1000 parabola from colorbanded males 2–3 years prior to conducting playbacks. The songs were recorded at 44.1 kHz sampling rate and stored as.wav files. To measure trill rate and bandwidth, we first resampled songs at 25 kHz and high pass filtered songs at 1500 Hz to remove noise below the range of NWCS songs. We then took trill minimum and maximum frequencies at −36 dB relative to the peak amplitude frequency from spectrograms (256 pt transform, frequency resolution: 97.7 Hz, 10.2 ms time resolution); this method captured variation in frequency bandwidth while excluding background noise^37^. We calculated frequency bandwidth as the difference between the maximum and minimum frequencies, and trill rate as the average number of trill notes produced per second. We collected all song data in Signal 5^52^. To calculate vocal performance, we used the published equation for the upper bound regression on a set of 1572 Emberzidae songs, y = −0.124 × + 7.55^37^. We calculated vocal performance as the orthogonal deviation of each song from this upper bound regression, hereafter referred to as vocal deviation. Vocal deviation is one of many ways to calculate performance^53–55^, and has been shown to be robust in Emberizids^56^.

**Figure 1:**
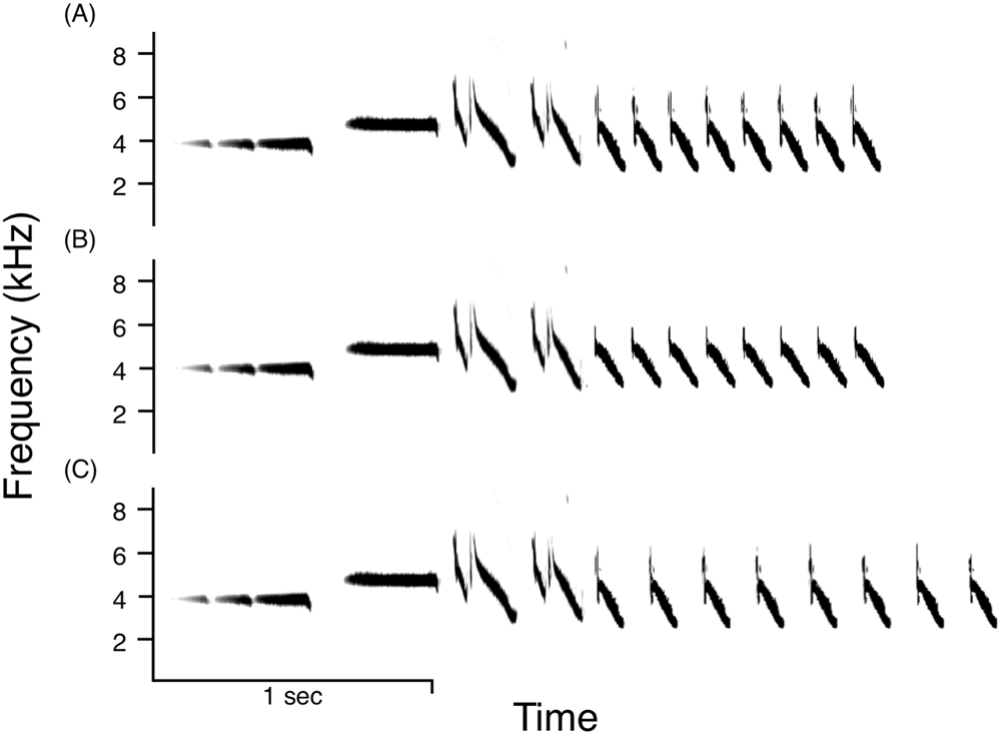
Example stimulus set of (**A**) fast trill, wide bandwidth song, (**B**) fast trill, narrow bandwidth song, and (**C**) slow trill, wide bandwidth song.

From the measured recordings described above, we created San Francisco dialect stimuli for song playback experiments^57^. Songs selected for stimuli had high signal to noise ratios. From the recordings, we drew pairs of songs that differed naturally by at least 500 Hz in trill bandwidth and then manipulated each song to create a slow and a fast trill version. To create specific trill rates, we repeated the first trill note eight times with the desired spacing between notes. We made stimulus sets that consisted of three song treatments: (A) wide bandwidth, fast trill rate, (B) narrow bandwidth, fast trill rate, and (C) wide bandwidth, slow trill rate (Fig. 1). We calculated the necessary slow and fast trill rate for each stimulus set such that stimuli ‘wide bandwidth, slow trill rate’ and ‘narrow bandwidth, fast trill rate’ would have roughly the same vocal performance value (t-test, t = 1.3, d.f. = 32, *p* = 0.2; Table 1). Amplitude is known to affect male response to playback in this species^58^, thus we normalized stimuli amplitude in SIGNAL 5^52^ and calibrated amplitude from the speaker at 1 meter to 81 dB with a Larson Davis 831 Sound Level Meter (PCB Piezotronics). All features of manipulated songs were within the normal range of songs for the San Francisco dialect^27^ (Table 1). We created 17 stimulus sets for trials based on the availability of high quality recordings with 500 Hz differences in bandwidth.

**Table 1:** A comparison of San Francisco songs and experimental stimuli for vocal deviation, trill rate, and bandwidth (range; mean ± SD).

Song Type	No. of songs	Vocal deviation	Trill rate (Hz)	Bandwidth (Hz)
SF dialect	780	5.3–37.4; 22.2 ± 5.2	6.1–13.3; 9.2 ± 1.2	1690.4–5735.4; 2753.2 ± 648.8
SF narrow bandwidth, fast trill stimuli	17	12.2–33; 23.9 ± 4.2	9.3–12.5; 11.4 ± 0.9	1975.7–4699.8; 3194.1 ± 563.8
SF wide bandwidth, slow trill stimuli	17	10.9–32.3; 22 ± 4.2	6.1–6.67; 6.3 ± 0.2	2768.7–5424.1; 4034.6 ± 515.8
SF wide bandwidth, fast trill stimuli	17	6.4–26.6; 16.7 ± 4	9.3–12.5; 11.4 ± 0.9	2768.7–5424.1; 4034.6 ± 515.8

Ranges for San Francisco (SF) dialect are from Phillips & Derryberry (2017a).

### Playback experiment design

**Figure 2:**
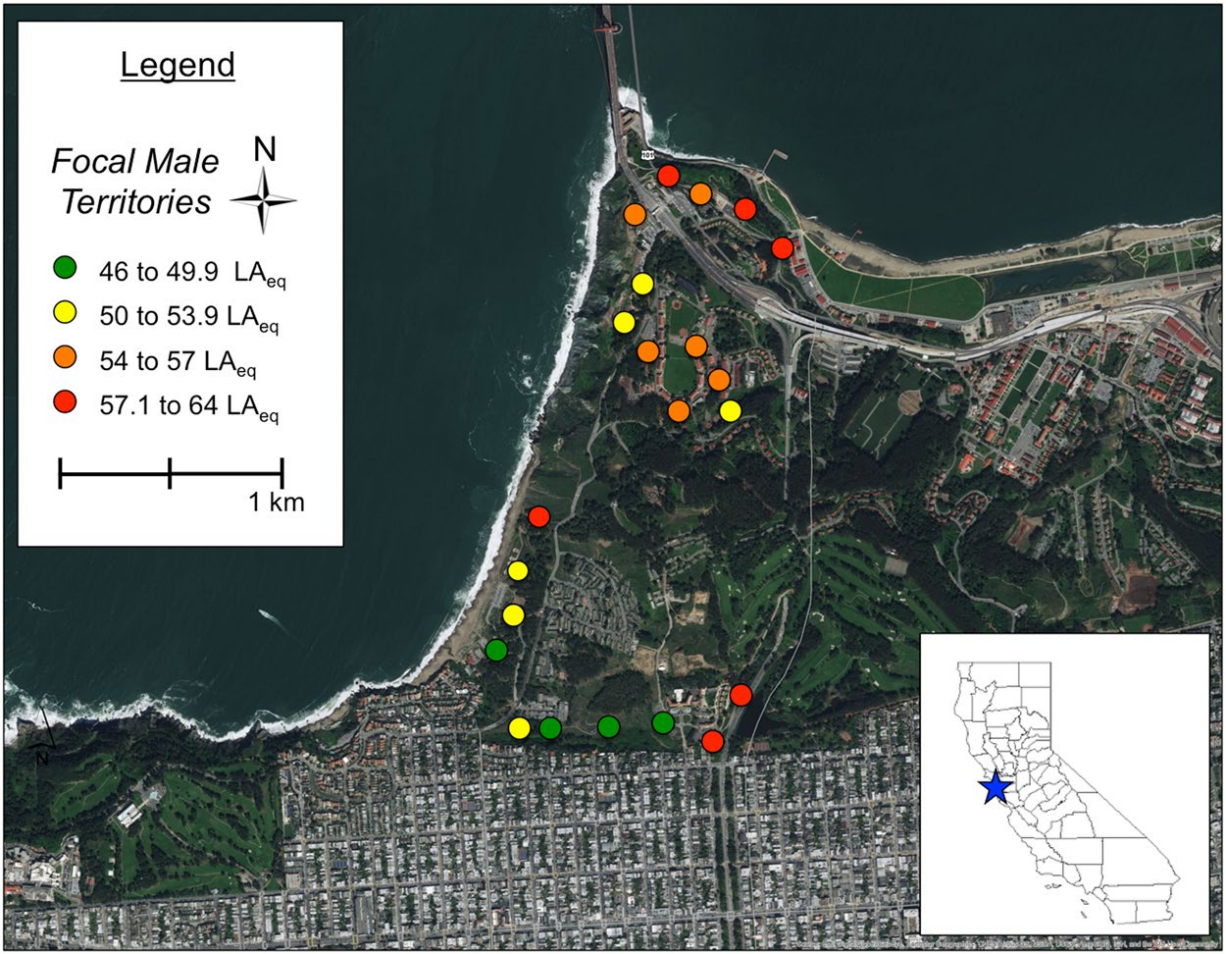
Map of focal male territories in the Presidio, San Francisco, California, USA. Circles denote an individual male and the average territory noise across three measurements. Map created in ArcMap 10 (ESRI, Redlands, CA, USA) with ESRI world imagery (Sources: ESRI, DigitalGlobe, GeoEye, Earthstar Geographics, CNES/Airbus DS, USDA, USGS, AeroGRID, IGN, and the GIS User Community).

We used repeated measures territorial playback experiments to test whether free-living adult males (*n* = 22) in urban environments responded differently to songs that varied in vocal performance and its component parts, trill rate and bandwidth. Territorial playback is a standard experimental design that simulates territorial intrusion by playing songs on subjects’ territories and measuring their behavioral response^59^. Subjects held territories in the Presidio of San Francisco (Golden Gate National Recreation Area) in the May 2016 breeding season (Fig. 2). Most males were colorbanded (*n* = 18), and all male territories were observed prior to playback to establish song perches and boundaries. Playbacks were conducted between sunrise and noon during the breeding season. We tested each male three times, once for each stimulus treatment; trials were conducted with at least 48 hours between trials to minimize habituation. Order of presentation was randomized across males. Neighbors were never tested on the same day, and we did not use songs from neighboring males as stimuli.

For each focal male, we observed song perches and determined the approximate location of the territory center. Before each trial, an inMotion iMT320 speaker (Altec Lansing) with an Apple iPod Nano (6^th^ generation) was placed near the territory center on a platform 0.5 m above the ground. The same location was used each time the male was tested. We started the playback when the focal male was in view within 24 m of the speaker to ensure he was on his territory. Once a trial began, songs were broadcast at a typical song bout speed (6 songs/min).

During each trial, we recorded the male’s movement behaviors at 10-sec intervals. We recorded responses during a three-minute playback period and a six-minute post-playback period. Post-playback was examined because responses tend to vary most between stimuli during the post-playback period in white-crowned sparrows^60^. The response variables therefore are latency to approach and approach distance to the speaker (m) during both playback and post-playback. We examine these two responses because approach distance is interpretable as likelihood to attack^59,61,62^ and latency to approach may serve as a proxy for an animal’s ability to detect a signal (following Kleist *et al*. 2016). To approximate distance measures, we placed a string radiating out from the speaker with distance categories marked with flagging tape. The distance categories used were 0–2 m, 2–4 m, 4–8 m, 8–16 m, and greater than 16 m. We used the median distance of each category and 24 m for the ‘greater than 16 m’ category to calculate the male’s average distance from the speaker during the playback and post-playback periods^62^.

Playback procedures were carried out in accordance with approved guidelines set by Tulane University Institutional Animal Care and Use Committee (protocol 0427-R), Bird Banding Laboratory Permit (23900), California State Collecting Permit (6799), Golden Gate National Recreation Area (GGNRA) Scientific Research and Collecting Permit (GOGA-00079), and San Francisco Parks and Recreation Permit (032014).

### Ambient noise level measurements

We measured ambient noise levels within five minutes of playback experiments using a Larson Davis 831 Class 1 Sound level meter (PCB Piezotronics). We took readings for one minute in each cardinal direction for a total of 4 minutes, following published methods^63^. Our values were recorded in LAeq, which accounts for noise fluctuations over time and adjusts for the range of audible noise for humans, which overlaps with that of songbirds^64^.

### Statistical analyses

To assess the effects of noise, stimulus treatment, and potential interactions between noise and stimulus treatment on approach distance during playback and post-playback, we explored all combinations of the fixed effects of stimulus and noise using linear mixed-effect models implemented in lme4^65^ and Akaike’s Information Criterion for small sample sizes, AIC_c_^66^. To examine directionality of response to fixed effects, we used post hoc Tukey t-tests for stimulus treatment and linear regression for territory noise. We examined the interaction between noise and stimulus because of our prediction that discrimination strength between stimuli would change with noise levels, which would result in different slopes for the relationship between noise and response for each stimulus treatment. We re-used 5 stimulus sets (stimulus sets: *n* = 17, total songs used as stimuli: *n* = 51; focal males: *n* = 22); thus, we included stimulus exemplar as a random effect in all models. Because males were tested with multiple stimuli in a repeated measures design, bird identity was also included as a random effect in all models. To examine relative variable importance, we averaged models within the 95% cumulative weight using MuMIn^67–69^. Response variables were log-transformed to meet model assumptions. To account for multiple comparisons in post-hoc tests, we use Bonferonni correction, with α = 0.017. We performed all statistical analyses in R^70^.

### Data availability

The datasets analyzed during the current study are available in the Supplemental Material.

## Results

### Stimulus treatment and territory noise level affect playback approach distance

**Figure 3:**
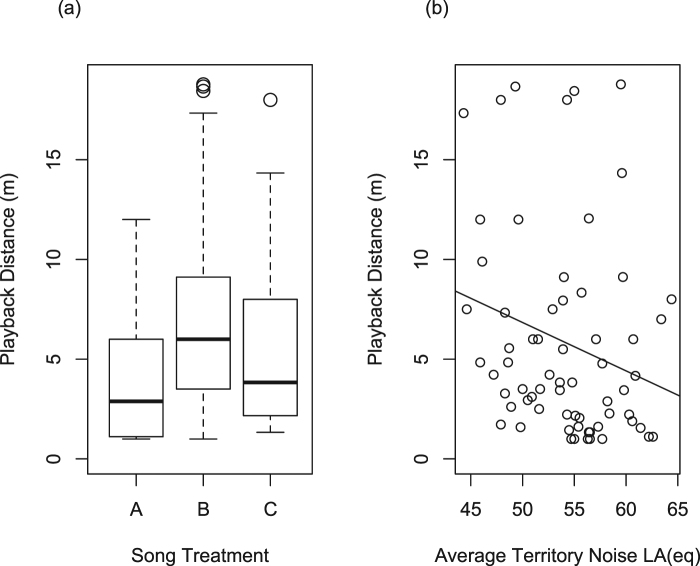
Playback approach distance results. (**a**) Boxplot of playback distance (m) response to three song treatments (A) fast trill, wide bandwidth (4 ± 0.74 m), (B) fast trill, narrow bandwidth (7.6 ± 1.22 m), and (C) slow trill, wide bandwidth (6 ± 1.14 m). A is significantly different than B (p < 0.001), but A & C and B & C are statistically equal (*p* > 0.05). (**b**) Linear regression of male approach distance during playback and territory noise showing males approach more closely during playback when it is noisy (β = −0.05, *p* = 0.015).

AIC_c_ model selection supports that stimulus treatment + territory noise predicts approach distance during the playback period (AIC_c_ = 164.4, weight = 0.46, ER = 39.56, Table 2). The next supported model within 2 AIC_c_ includes only stimulus treatment (Table 2). A model average of the three models within the 95% cumulative weight^69^ shows stimulus treatment with a relative weight of 100%, territory noise level with a relative weight of 65%, and the interaction between the two has a relative weight of 16%. Post-hoc tests show playback approach distance is significantly closer to fast trill, wide bandwidth songs than to fast trill, narrow bandwidth songs (Tukey’s t-test: z = 3.47, *p* < 0.001) but not to slow trill, wide bandwidth songs (Tukey’s t-test: z = 1.78, *p* = 0.17; Fig. 3a). Males did not differ in their response to songs of similar vocal performance (i.e., fast trill, narrow bandwidth and slow trill, wide bandwidth songs; Tukey’s t-test: z = −1.67, *p* = 0.21; Fig. 3a). Post-hoc analysis also showed that males approached the playback speaker more closely as territory noise level increased across treatments (β = −0.05, R^2^ = 0.09, F_1, 64_ = 6.24, *p* = 0.015; Fig. 3b), but discrimination strength between high and low performance songs did not change with average territory noise (β = 0.2, R^2^ = 0.03, F_1, 42_ = 1.6 *p* = 0.2).

**Table 2:** AIC_c_ table for playback distance.

Models	K	AIC_c_	ΔAIC_c_	w_i_	Cumulative w_i_
Stimulus + Noise	7	164.40	0	0.47	0.47
Stimulus	6	165.03	0.63	0.34	0.81
Stimulus + Noise + Stimulus*Noise	9	166.63	2.23	0.15	0.96
Noise	5	170.05	5.64	0.03	0.99
Null	4	171.76	7.36	0.01	1

### Noise affects post-playback approach distance

**Figure 4:**
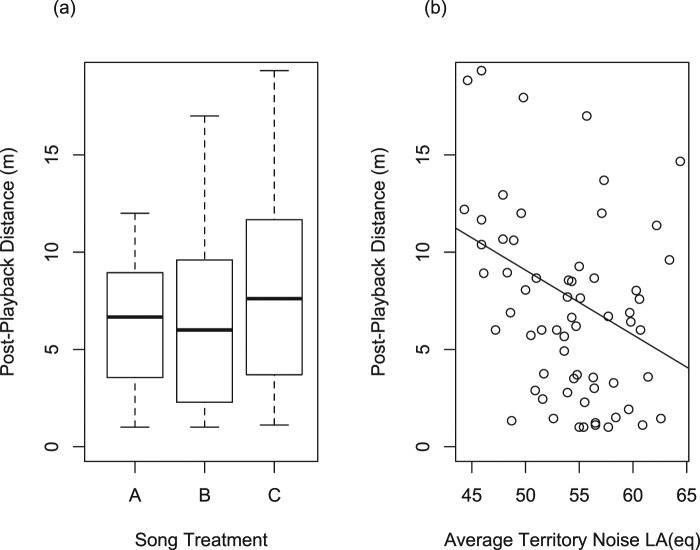
Post-playback approach distance results. (**a**) Boxplot of approach distance for post-playback for three stimulus treatments: (A) fast trill, wide bandwidth (7.11 ± 0.96 m), (B) fast trill, narrow bandwidth (7.54 ± 1.41 m), and (C) slow trill, wide bandwidth (8.54 ± 1.19 m). All post-hoc comparisons between treatments *p* > 0.05. (**b**) Linear regression of male approach distance during post-playback and territory noise (β = −0.05, *p* = 0.012).

AIC_c_ model selection supports that territory noise predicts approach distance during the post-playback period (AIC_c_ = 165.49, weight = 0.71, ER = 6.45, Table 3). Model averaging within the 95% cumulative weight shows the relative weight of territory noise across models was 88% and for stimulus treatment was 15%. Tukey’s post-hoc comparison of stimulus treatments showed no significant differences between all three treatments (Fig. 4a; all *p* > 0.05). A post-hoc linear regression shows territory noise to be a significant predictor of male response, with males approaching more closely as noise increases (β = −0.05, R^2^ = 0.095, F_1, 64_ = 6.17, *p* = 0.012; Fig. 4b).

**Table 3:** AIC_c_ model selection for post-playback approach distance.

Model	K	AIC_c_	ΔAIC_c_	w_i_	Cumulative w_i_
Noise	5	165.49	0	0.71	0.71
Stimulus + Noise	7	168.74	3.24	0.14	0.85
Null	4	169.19	3.69	0.11	0.96
Stimulus	6	172.08	6.58	0.03	0.99
Stimulus + Noise + Stimulus*Noise	9	173.60	8.11	0.01	1

### Males tend to approach more quickly in noise

The top model for latency to approach had a fixed effect of territory noise (AIC_c_ = 176.98, weight = 0.65, ER = 2.9), followed by the null model (AIC_c_ = 179.11, weight = 0.22). A Type II ANOVA of the top model was significant (*x*^2^ = 7.9, d.f. = 1, *p* = 0.03). Tukey’s post-hoc comparison of stimulus treatments showed no significant differences between all three treatments in latency to approach (all *p* > 0.05). A post-hoc linear regression shows territory noise weakly predicts latency to approach, with males tending to approach more quickly as noise increases (β = −0.3, R^2^ = 0.05, F_1, 64_ = 3.65, *p* = 0.06).

## Discussion

Overall, we find that urban male NWCS approach more closely to wide bandwidth than to narrow bandwidth songs, but they do not respond differently to fast and slow trills. These results are opposite of our predictions. Consistent with our predictions of a more aggressive response in noise, we found that territory noise level may affect response distance, especially during post-playback. As noise increases, males tend to approach more closely to stimulus songs during playback, and remain close or move closer after playback no matter which stimulus was played. We found weak support for a predicted interaction between stimulus treatment and territory noise levels during the playback period, and no support for an interaction during the post-playback period. These results suggest no decrease in discrimination strength between stimulus types in noisy conditions. Together, our findings indicate that urban males assess variation in bandwidth but not in trill rate, and that males defending noisier territories may be more likely to face costly fights, because a closer approach to an intruding male increases the probability of attack^61^.

We find partial support for our hypothesis that noise-dependent adjustments of bandwidth and trill rate in urban populations have functional consequences. Specifically, males approached more closely to wide bandwidth songs than to narrow bandwidth songs during playback. A closer approach to a speaker in a male’s territory is interpreted as a stronger response to that stimulus; thus, males respond more strongly to wide bandwidth songs. This finding is consistent with previous studies testing response to variation in song bandwidth in both urban and rural males^28,43^, and supports the hypothesis that males producing narrower bandwidth songs have less potent signals in the context of male-male competition. Male white-crowned sparrows that defend noisier territories produce songs with significantly narrower bandwidth than males that defend quieter territories (Luther *et al*. 2016b). This trend is found in many urban species, such as song sparrows (*Melospiza melodia*)^71^, dark-eyed juncos (*Junco hyemalis*)^72^, great tits (*Parus major*)^73^, European robins (*Erithacus rubecula*)^74^, chipping sparrows (*Spizella passerina*)^20^, cardinals (*Cardinalis cardinalis*) and catbirds (*Dumetella carolinensis*)^19^. Thus, noise-dependent shifts in bandwidth have consequences for NWCS and potentially for other songbird species, at least in the context of territory acquisition and maintenance.

We did not find support for adjustments to trill rate affecting male response. Male NWCS defending noisier territories produce songs with faster trill rates than males on quieter territories^28^. Our experimental results here indicate that males respond similarly to fast trill and slow trill songs. Thus, an increase in trill rate on noisier territories in San Francisco does not appear to have a strong effect on male-male competition in this location. In contrast, in a previous study we found that males in nearby areas outside of the city limits of San Francisco (in Marin County) do respond to variation in trill rate^43^, such that males respond less to slow trills than to fast trills when bandwidth is consistent. Furthermore, studies in other species, like swamp sparrows (*Melospiza georgiana*) and banded wrens (*Thryothorus pleurostictus*) found that males typically increase trill rate to indicate aggressive motivation^75,76^, and males assess differences in trill rate^29,77^. One reason for the lack of responsiveness to variation in trill rate in the city may be that slow trills transmit better in the city, even in areas that are not obvious ‘urban canyons’, like the Presidio of San Francisco. If this is the case, then males may be responding less to fast trills in urban areas because the fast trills do not transmit as far, which leads to an equal response to fast and slow trill rates. This finding is similar to that in great tits in which females respond less to typically potent low frequency songs during noisy times of day because low frequency songs do not transmit as well^18^. Although our previous work indicates that songs with higher minimum frequency and narrower bandwidth transmit over greater communication distances in urban areas^78^, we do not know if trill rate affects transmission in city noise in this species. Further studies would need to test how trill rate affects communication distance in these areas.

We also found no significant difference in response to songs of similar vocal deviation, similar to results found in a rural population of NWCS^27^. Thus, finding an equal response to these two types of stimuli would seem to suggest no functional difference among the songs produced by males holding territories with different ambient noise levels. However, it is important to note that despite the increase in trill rate on noisier territories, urban males still produce songs of lower performance^28^. Males do tend to approach more closely to wide bandwidth, slow trill songs than narrow bandwidth, fast trill songs (Fig. 2). Our playback experiment indicates that male receivers respond less to songs of lower performance, supporting that songs more typical of NWCS defending noisier territories are less potent than those of males defending quieter territories, on average.

Our hypothesis that the functional consequences of noise-dependent song adjustments vary with ambient noise levels was partially supported. We predicted that the strength of response to variation in vocal performance would decrease with increasing levels of ambient noise and that overall level of response to stimulus playback would increase. This can also be described as a ceiling effect such that as response increases to all stimuli, the difference in response to different stimuli will decrease. Although we did find a significant increase in the level of response to all stimuli with increasing levels of noise, we did not find a ceiling effect. There was no interaction between song treatment and ambient noise levels, indicating that response slopes did not vary among the song treatments. In other words, males are responding less to narrow bandwidth songs on both quiet and noisier territories, not just less on noisier territories.

We found that males on noisier territories come closer to all stimulus song types than do males on quieter territories. One interpretation of this result is that the urban environment, with particularly high levels of noise, may lead to overall higher aggression levels in cities, or so-called ‘urban anger’. Increased aggression has been observed in urban birds^46,47,49^, typically as measured by approach distance. However, the cause of urban anger has been elusive – studies have not found support for higher population density, available nesting habitat, or testosterone levels as predictors of aggression levels in urban males^49,50^, although one study finds some support for food availability as a driver of urban aggression^49^. We find that males on noisier territories approach more closely to simulated territory intrusions, and so this finding suggests that territory noise levels may also be a factor in increased levels of aggression in urban areas. It may be that males on noisy territories have a decreased response threshold caused by acoustic masking, which leads to unnecessary or inappropriately strong responses^79,80^. Future experimental studies could test the effects of chronic noise on aggression levels, and if detection threshold patterns are similar in NWCS as in species previously tested^13,14,81^.

Another interpretation of the finding that males approach songs more closely in noise is that males on noisier territories cannot detect song playbacks as readily as males on quieter territories. Detection is the ability of a bird to hear a sound at a certain distance whereas discrimination is the ability of a bird to tell the difference between sounds, or identify their characteristics^13^. However, if males on noisier territories could not detect song playbacks as quickly as males on quieter territories, then we would expect a longer latency to approach in noise (e.g. Luther & Magnotti 2014; Kleist *et al*. 2016). Instead, we find that males on noisier territories tend to have a shorter latency to approach. In other words, males approach playbacks more quickly in noise. Thus, our findings suggest that males on noisier territories can detect song playbacks as readily as males on quiet territories but may not be able to discriminate fine features of the song without approaching more closely. Thus, our results suggest that to assess song performance, males may have to approach more closely to enter the active space or listening distance of the signal bandwidth^13,82^. If a male cannot assess an intruding male without getting closer, both are more likely to incur a physical cost.

Lower performance songs have a greater communication distance in noise but are less salient in territorial intrusions. Some animals may be able to use tactical allocation to minimize this apparent cost of signaling in noise. Selection should favor males to be flexible within their performance range, such that they sing at their performance limit only when a female or intruder is nearby (thus reducing the communication distance and associated effects of sensory drive). When territorial males are not contending with nearby intruders or potential mates, producing a song with a lower performance value that transmits further may be beneficial, as increasing communication distance may outweigh any performance costs^83^. This type of tactical allocation falls under the ‘Maximizing Received Signal Hypothesis’^84^. For example, house finches (*Haemorhous mexicanus*) display some syllable plasticity in pitch (Bermudez-Cuamatzin *et al*. 2008) and white-crowned sparrows show immediate flexibility in song production by varying number of trill notes based on motivation levels^85^. However, a recent study shows male white-crowned sparrows do not change song pitch in real time in response to changing levels of noise^86^, suggesting that they may not be able to adjust song bandwidth. However, if males in this species exhibit immediate flexibility in trill rate, then tactical allocation might be a solution to conflicting selection pressures from urban noise and male-male competition.

Although we have demonstrated that males holding territories with high ambient noise levels may bear a cost in singing lower performance songs in the context of male-male competition, we do not know what costs they may face in the context of female mate choice. Females in other songbirds prefer high performance songs^31^, so urbanization of song may also affect female mate choice. It remains to be seen if female choice is affected by noise in NWCS. One study suggests that great tit females may respond less to high-pitched songs that have narrower bandwidth (i.e., lower performance songs; Halfwerk *et al*. 2011). Masking of songs has also been shown to alter female preference for a pair-bonded mate^87^. Males may be able to negate a loss of female preference in noise by singing louder, moving closer to females, or by enhancing their performance via bandwidth when females are most fertile^18^. Future studies examining whether preferences for song change in noisy anthropogenic conditions are essential.

## Conclusions

Our study highlights a functional consequence of song modification in urban landscapes, particularly for birds with trilled vocalizations. Additionally, our study is the first to show that assessment of vocal performance, a known sexually selected trait, is at least partially affected by ambient noise levels. We found that males approach simulated intruders more closely in noisier conditions, suggesting an increase in the chance for territorial disputes as males assess sexual signals. Closer approach in noise may indicate increased aggression or the need for receivers to enter the active space of the signal to discriminate song characteristics—disentangling these two interpretations is an open field of inquiry. Future research is needed to examine these consequences in the context of other performance measures of song (song rate, repertoire size, amplitude) in additional species and across a wider range of soundscapes. As anthropogenic soundscapes become more the norm than the exception, understanding the impacts humans have on animal communication is critical.

## Electronic supplementary material

Supplementary Dataset 1
